# Short-term OS as a surrogate endpoint for 5-year OS in nasopharyngeal carcinoma in non-endemic area

**DOI:** 10.1186/s12957-024-03460-z

**Published:** 2024-07-11

**Authors:** Ying Guan, Lu Han, Han-Yin Luo, Bin-Bin Yu, Shi-Ting Huang

**Affiliations:** 1 Department of Radiation Oncology, Guangxi Medical University Cancer Hospital, Nanning, Guangxi 530021 China; 2 Department of Oncology, Wuming Hospital of Guangxi Medical University, Nanning, Guangxi 530199 China

**Keywords:** Surrogate endpoint, Nasopharyngeal carcinoma, Short-term endpoints, Overall survival, SEER

## Abstract

**Purpose:**

To address this evidence gap and validate short-term OS at less than 5 years as a reliable surrogate endpoint for 5-year OS.

**Methods:**

We analyzed data from the Surveillance, Epidemiology, and End Results (SEER) database, focusing on non-metastatic NPC patients diagnosed between 2010 and 2015. Patients were categorized into radiotherapy and chemoradiotherapy groups.

**Results:**

This retrospective study examined 2,047 non-metastatic NPC patients. Among them, 217 received radiotherapy, and 1,830 received chemoradiotherapy. Our analysis results indicated that the 4-year OS may serve as a reliable surrogate endpoint for patients with AJCC clinical stage I (80 vs. 78%, *P* = 0.250), regardless of the treatment received. Specifically, in the radiotherapy group, patients with stage I, T0-T1, and N0 NPC showed similar OS rates at 4 and 5 years (83 vs. 82%, *P* = 1.000; 78 vs. 76%, *P* = 0.250; 78 vs. 77%, *P* = 0.500, respectively). Similarly, patients with stage II-IV, T2-T4, and N1-3 NPC showed no significant difference in OS rates between 3 and 5 years (57 vs. 51%, *P* = 0.063; 52 vs. 46%, *P* = 0.250; 54 vs. 46%, *P* = 0.125, respectively) in the radiotherapy group. In the chemoradiotherapy group, only the 3-year OS rate did not significantly differ from that at 5 years in stage I patients (79vs. 72%, *P* = 0.063).

**Conclusions:**

Our study suggests that short-term surrogate endpoints may be valuable for evaluating 5-year OS outcomes in NPC patients in non-endemic areas.

**Supplementary Information:**

The online version contains supplementary material available at 10.1186/s12957-024-03460-z.

## Introduction

Nasopharyngeal carcinoma (NPC) is a malignancy affecting the nasopharyngeal epithelium [1]. It displays sensitivity to radiotherapy and chemotherapy [2]. For stage I NPC, radiotherapy alone is the recommended treatment [3]. However, NPC is often diagnosed at advanced stages, making concurrent chemoradiotherapy the standard of care for patients with locally advanced NPC. In the United States, NPC is relatively rare, with an incidence of less than 1 per 100,000 individuals [4]. Despite its rarity, the 5-year overall survival (OS) rate of 60% [5] is concerning, and recent trends indicate comparable incidence rates to those observed in endemic regions such as Southeast Asia [6]. Understanding the endemic nature and survival disparities remains essential for clinicians, given the tumor’s heterogeneity [7].

In oncology clinical trials, the ultimate goal is to improve long-term outcomes and increase cure rates. As a result, the five-year overall survival (OS) has long been the gold standard endpoint due to its simplicity and patient-friendliness. Nevertheless, its drawback lies in the requirement for prolonged follow-up. To address this, researchers have introduced surrogate endpoints, representing time points that closely and consistently correlate with the 5-year OS but are reached more rapidly. These surrogate endpoints offer numerous advantages, including faster trial results and potentially quicker drug approval and availability. For practical use, an ideal surrogate endpoint should not only predict treatment effects on the actual endpoint but also be relevant, understandable, and easy to explain to patients.

The incidence of NPC is significantly influenced by regional characteristics, with Southern China reporting the highest rates [1, 8]. China has made considerable progress in NPC OS over the years, owing to improvements in radiotherapy and widespread application of chemotherapy [9, 10]. The 5-year OS rate ranges from 65% for locally advanced disease to 90% for early-stage cases [11]. Studies and meta-analyses on surrogate endpoints (summarized in Supplementary Table S1) support the use of short-term OS and other survival endpoints (such as loco-regional control [LRC], progression-free survival [PFS], distant metastasis-free survival [DMFS], loco-regional recurrence-free survival [LRFS]) of less than five years as surrogates. However, the reliability and adequacy of other survival endpoints as surrogates for OS may depend on the recurrence/metastasis pattern and available salvage treatment options. Significant differences in other survival endpoints might translate into the benefits of OS in the adjuvant cytotoxic chemotherapy setting, considering the assumption that micrometastatic disease is eliminated by the adjuvant treatment. However, the availability of more effective salvage treatments for recurrent or metastatic disease in recent times could weaken the association between recurrence/metastases-free survival and OS by improving survival after recurrence or metastases [2, 12], leading to no apparent differences in OS [13]. Therefore, ongoing re-evaluation of surrogate endpoints is crucial, particularly in the context of rapidly evolving therapeutic standards and changing assumptions guiding their use. Nonetheless, OS remains the preferred and routine primary endpoint.

Given substantial geographical disparities in NPC, it remains uncertain whether OS at less than 5 years can replace the 5-year follow-up in non-endemic areas for non-metastatic NPC patients. For surrogate endpoints to be valid, changes in these endpoints resulting from different treatment interventions should reliably translate to changes in true clinical endpoints. Therefore, our study aimed to address this evidence gap and validate short-term OS at less than 5 years as a reliable surrogate endpoint for 5-year OS.

## Materials and methods

### Data source, patient populations, and variables selection

In this retrospective study, our goal was to investigate whether short-term OS at less than 5 years could serve as a surrogate for 5-year OS in non-metastatic NPC in non-endemic areas. To achieve this, we utilized data from the Surveillance, Epidemiology, and End Results (SEER) program of the National Cancer Institute (https://seer.cancer.gov/data/).

We collected data for 3,536 NPC patients diagnosed between 2010 and 2015 from the SEER*Stat software (version 8.4.1): Incidence-SEER Research Plus Data, 18 Registries, Nov 2020 Sub (2000–2018) (Table 1), which included population-based data from 18 cancer registries covering approximately 27.8% of the United States (U.S.) population between 2000 and 2018. For inclusion in the study, patients had to meet specific criteria: (1) pathologically confirmed NPC; (2) limited histological subtypes according to the World Health Organization (WHO) classification scheme ICD-O-3(International Classification of Diseases for Oncology, 3rd Edition), including WHO I (keratinizing squamous cell carcinoma, KSCC, ICD-O-3 codes: 8070 and 8071), WHO II (differential non-keratinizing carcinoma, DNKSCC, ICD-O-3 codes: 8072 and 8073), WHO III (undifferentiated non-keratinizing carcinoma, UNKSCC, ICD-O-3 codes: 8020, 8021, and 8082), and others(ICD-O-3 codes: 8083 and 8010) [14, 15]; (3) NPC patients without metastasis (M0) diagnosed according to the American Joint Commission on Cancer (AJCC) 7th staging system; (4) patients who received radiotherapy, with the exclusion of simple radioactive implantation; (5) NPC being the first and only primary malignancy. Exclusion criteria consisted of (1) patients who did not receive any treatment; (2) patients with metastasis; (3) missing or unknown survival data; (3) follow-up duration less than 30 days; (4) data on T stage, N stage, M stage, stage groups, and race not recorded; (5) patients with non-first or not only one primary malignant tumor. No written informed consent is required since identifiable information on individual patients was not utilized in this study.

**Table 1:** Patients baseline characteristics

Variables (%)	Total *N* = 2047	Radiotherapy *N* = 217	Chemoradiotherapy *N* = 1830
**Age (%)**			
Median (Range)	54(7–92)	59(17–92)	54(7–89)
≤ 54years	1053 (51.4)	82 (37.8)	971 (53.1)
> 54years	994 (48.6)	135 (62.2)	859 (46.9)
**Sex (%)**			
Male	1434 (70.1)	151 (69.6)	1283 (70.1)
Female	613 (29.9)	66 (30.4)	547 (29.9)
**Race (%)**			
White	893 (43.6)	96 (44.2)	797 (43.6)
Black	238 (11.6)	28 (12.9)	210 (11.5)
Others†	916 (44.7)	93 (42.9)	823 (45.0)
**Marriage (%)**			
Married	1493 (72.9)	163 (75.1)	1330 (72.7)
Unmarried	441 (21.5)	39 (18.0)	402 (22.0)
Unknown	113 (5.5)	15 (6.9)	98 (5.4)
Grade (%)*			
I-II	195 (9.5)	23 (10.6)	172 (9.4)
III	601 (29.4)	60 (27.6)	541 (29.6)
IV	633 (30.9)	70 (32.3)	563 (30.8)
Unknown	618 (30.2)	64 (29.5)	554 (30.3)
**Histology (%)**			
WHO I	681 (33.3)	75 (34.6)	606 (33.1)
WHO II	594 (29.0)	58 (26.7)	536 (29.3)
WHO III	387 (18.9)	40 (18.4)	347 (19.0)
Others§	385 (18.8)	44 (20.3)	341 (18.6)
**Stage T (%)**			
T0‡-T1	725 (35.4)	136 (62.7)	589 (32.2)
T2	415 (20.3)	34 (15.7)	381 (20.8)
T3	416 (20.3)	23 (10.6)	393 (21.5)
T4	491 (24.0)	24 (11.1)	467 (25.5)
**Stage N (%)**			
N0	433 (21.2)	135 (62.2)	298 (16.3)
N1	686 (33.5)	40 (18.4)	646 (35.3)
N2	636 (31.1)	25 (11.5)	611 (33.4)
N3	292 (14.3)	17 (7.8)	275 (15.0)
**TNM stage (%)**			
I	169 (8.3)	100 (46.1)	69 (3.8)
II	466 (22.8)	41 (18.9)	425 (23.2)
III	674 (32.9)	37 (17.1)	637 (34.8)
IV	738 (36.1)	39 (18.0)	699 (38.2)
**Tumor size (%)**			
≤ 3 cm	450 (22.0)	76 (35.0)	374 (20.4)
> 3 cm	858 (41.9)	62 (28.6)	796 (43.5)
Unknown	739 (36.1)	79 (36.4)	660 (36.1)
**Lymph node size (%)**			
0 cm	410 (20.0)	130 (59.9)	280 (15.3)
≤ 2 cm	442 (21.6)	19 (8.8)	423 (23.1)
> 2 cm	748 (36.5)	37 (17.1)	711 (38.9)
Unknown	447 (21.8)	31 (14.3)	416 (22.7)
**Primary site (%)**			
Superior wall	27 (1.3)	3 (1.4)	24 (1.3)
Posterior wall	267 (13.0)	37 (17.1)	230 (12.6)
Lateral wall	188 (9.2)	27 (12.4)	161 (8.8)
Anterior wall	20 (1.0)	5 (2.3)	15 (0.8)
Overlapping wall	80 (3.9)	4 (1.8)	76 (4.2)
Unknown	1465 (71.6)	141 (65.0)	1324 (72.3)
**Surgery to primary site (%)**			
NO	1832 (89.5)	180 (82.9)	1652 (90.3)
Yes	215 (10.5)	37 (17.1)	178 (9.7)
**Surgery to lymph node (%)**			
NO	1531 (74.8)	186 (85.7)	1345 (73.5)
Yes	516 (25.2)	31 (14.3)	485 (26.5)
**All cause death (%)**			
Alive	1362 (66.5)	144 (66.4)	1218 (66.6)
Death	685 (33.5)	73 (33.6)	612 (33.4)
**Follow up median (Rang), months**			
	53(1-107)	55(1-107)	53(1-107)

*Abbreviations*: Others†, American Indian, Alaska Native, Asian, Pacific Islander; Others§: ICD-O-3 (International Classification of Diseases for Oncology, 3rd Edition) codes: 8083 and 8010, according to the World Health Organization (WHO) classification scheme; T0‡, Epstein-Barr virus (EBV) positive-unknown primary cancer with cervical lymph node involvement. Overall stage [TNM] stage, primary tumor [T] stage, lymph node [N] status according to American Joint Commission on Cancer (AJCC) 7th staging system; Grade*: According to SEER research plus data description cases diagnosed in 1975–2017, grading and differentiation codes are defined in ICD-O-2(International Classification of Diseases for Oncology, 2nd Edition). The description as follows: Grade I: well differentiated; differentiated, NOS; Grade II: moderately differentiated; moderately differentiated; intermediate differentiation; Grade III: poorly differentiated; differentiated; Grade IV: undifferentiated; anaplastic

We extracted various data variables, including patient ID, year of diagnosis, histologic type ICD-O-3, Grade, T stage, N stage, M stage, stage groups, radiotherapy, chemotherapy, survival months, vital status recode, and SEER cause-specific death classification, from the SEER database.

### Treatments and endpoint

Patients were divided into two groups: radiotherapy and chemoradiotherapy groups. The radiotherapy group received only radiotherapy, while the chemoradiotherapy group received radiotherapy and chemotherapy. Unfortunately, specific details about chemotherapy and radiotherapy were not available in the SEER database [16, 17]. The OS we analyzed was defined in the SEER base as the time from diagnosis until death from any cause. We established the 1-, 2-, 3-, 4-, and 5-year OS endpoints using the cause of death (COD) and survival time.

The OS rates of the three patient groups (total, chemoradiotherapy, and radiotherapy groups) at 4 and 5 years were compared. The analysis results demonstrated that the stage of the tumor had a notable impact on survival outcomes. Consequently, we conducted separate analyses in all three populations for patients stratified by the AJCC 7th staging system. For groups where the 4- and 5-year OS rates did not significant differ, additional analysis was performed by calculating the 1-, 2-, and 3-year OS rates which were then compared with the 5-year OS rate. The survival rate in the last comparison that showed no significant difference was then considered the surrogate endpoint for the 5-year OS.

### Statistical analysis

The life table technique was utilized for computing survival rates. The Kaplan-Meier method along with the two-sided log-rank test for visualization of survival curves to assess group differences. McNemar’s test was utilized for comparing the survival rates. Continuous variables were analyzed using Student’s t-test to identify differences, while categorical variables were compared using either the Chi-squared test or Fisher’s exact test. All statistical tests were two-tailed and *P* < 0.05 indicated a statistically significant difference. Statistical analyses were performed using RStudio software (version 4.2.1) and SPSS (version 26.0).

## Results

### Patient characteristics

In this study, we enrolled a total of 2,047 eligible patients, with a median follow-up duration of 53 months (ranging from 1 to 107 months). Age was categorized based on the median value, and tumor size and lymph node size were divided into three groups (< 3, ≥3 cm, and unknown; 0 cm, ≤2 cm, and > 2 cm, separately) using recognized cutoff values. able 1 summarized the baseline demographic and disease characteristics of the patients, where stage I NPC patients accounted for 8.3% (169) of the total group. The most common histologic types were WHO I (33.3%) and WHO II (29%). Within the radiotherapy group, we observed 46.1% (100) of patients with stage I NPC, 18.9% (41) with stage II NPC, 17.1% (37) with stage III NPC, and 18% (39) with stage IV NPC. Conversely, the chemoradiotherapy group had 3.8% (69) of patients in stage I, 23.2% (425) in stage II, 34.8% (637) in stage III, and 38.2% (699) in stage IV.

### Five-year overall survival surrogate endpoints

Among the 2,047 patients with non-metastatic NPC, 217 underwent radiotherapy and 1,830 received chemoradiotherapy. To address the limited sample size regarding clinical stages in the radiotherapy group, we further divided the patients into specific subgroups for analysis based on the AJCC clinical stage, T stage, and N stage: stage I and II-IV groups, T0-T1 and T2-T4 groups, N0 and N1-3 groups. The 5-year OS rates were 65% for total patients, 65% for radiotherapy, and 64% for the chemoradiotherapy group. Tables 2 and 3 displays the annual survival rates and P-values for comparing different years with the earliest year of the surrogate OS endpoint.

**Table 2:** Annual survival rates and their comparisons of overall survival (OS) in different groups of patients

Stratification			OS (%)					*P*-value			
			1year	2year	3year	4year	5year	4- vs. 5-year OS	3- vs. 5-year OS	2- vs. 5-year OS	1- vs. 5-year OS
Total (*N* = 2047)	Total		83	77	73	68	65	0.001	NA	NA	NA
	TNM stage	I	93	88	84	80	78	**0.250**	0.001	NA	NA
		II	90	85	82	78	76	0.001	NA	NA	NA
		III	84	78	74	69	65	0.001	NA	NA	NA
		IV	76	69	64	59	53	0.001	NA	NA	NA
	Stage T	T0-T1	88	82	80	76	74	0.000	NA	NA	NA
		T2	89	84	79	73	69	0.000	NA	NA	NA
		T3	79	71	66	61	58	0.000	NA	NA	NA
		T4	74	68	63	58	52	0.000	NA	NA	NA
	Stage N	N0	83	78	74	70	68	0.004	NA	NA	NA
		N1	86	80	77	71	68	0.000	NA	NA	NA
		N2	83	77	72	69	64	0.000	NA	NA	NA
		N3	76	67	63	57	53	0.000	NA	NA	NA
Radiotherapy (*N* = 217)	Radiotherapy		79	73	71	68	65	**0.063**	0.001	NA	NA
	TNM stage	I	95	91	87	83	82	1.000	0.031	NA	NA
		II-IV	66	58	57	54	51	0.125	**0.063**	0.000	NA
	Stage T	T0-T1	90	85	83	78	76	0.250	0.008	NA	NA
		T2-T4	60	54	52	51	46	0.500	**0.250**	0.008	NA
	Stage N	N0	88	84	82	78	77	0.500	0.016	NA	NA
		N1-N3	64	55	54	50	46	0.250	**0.125**	0.001	NA
Chemoradiotherapy (*N* = 1830)	Chemoradiotherapy		84	77	73	68	64	0.000	NA	NA	NA
	TNM stage	I	90	84	79	75	72	0.500	**0.063**	0.004	NA
		II	90	86	82	78	76	0.002	NA	NA	NA
		III	85	78	75	70	66	0.000	NA	NA	NA
		IV	78	71	65	60	55	0.000	NA	NA	NA
	Stage T	T0-T1	88	81	79	76	73	0.002	NA	NA	NA
		T2	89	85	79	73	69	0.000	NA	NA	NA
		T3	80	73	68	62	59	0.000	NA	NA	NA
		T4	77	70	65	60	54	0.000	NA	NA	NA
	Stage N	N0	81	75	71	67	64	0.016	NA	NA	NA
		N1	87	82	78	73	70	0.000	NA	NA	NA
		N2	84	78	73	69	64	0.000	NA	NA	NA
		N3	78	69	64	58	54	0.000	NA	NA	NA

**Table 3:** Year of surrogate endpoints in different groups stratified by TNM staging

AJCC 7th staging	Year of surrogate endpoints for 5-year overall survival (OS)
Total	
I	4
Radiotherapy	
I	4
II-IV	3
T0-T1	4
T2-T4	3
N0	4
N1-N3	3
Chemoradiotherapy	
I	3

*Abbreviation* AJCC: American Joint Commission on Cancer

**Fig. 1:**
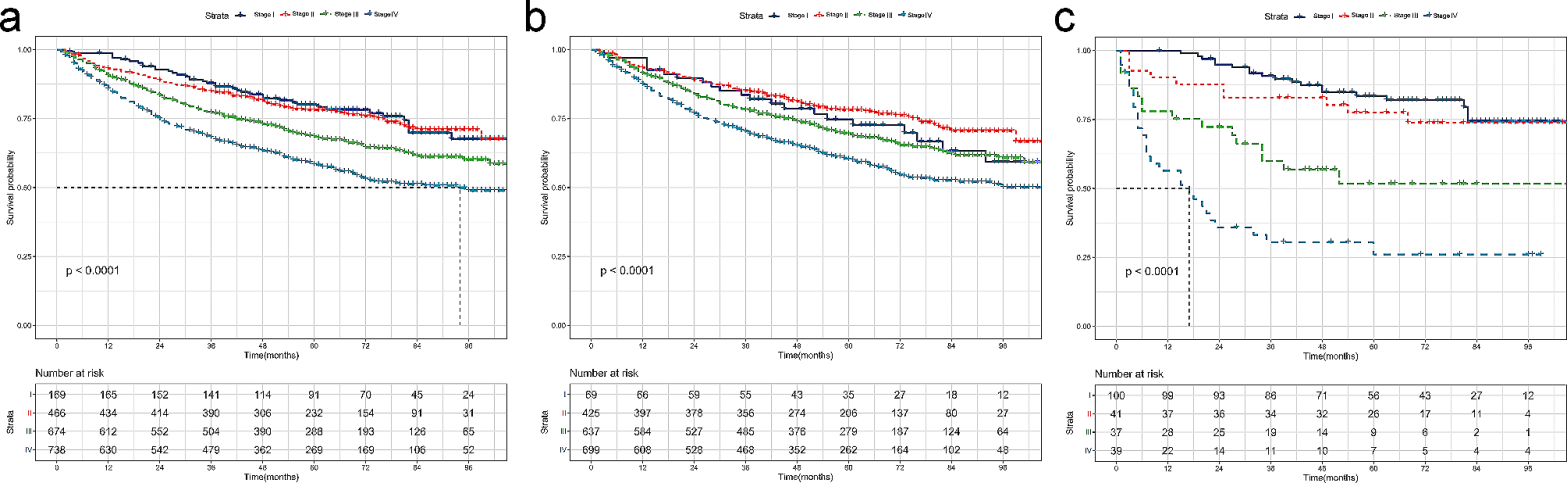
Overall Survival (OS) curves stratified by the American Joint Commission on Cancer (AJCC) 7th staging system for three groups. (**a**) total group stratified by stages I-IV; (**b**) chemoradiotherapy group stratified by stages I-IV; and (**c**) radiotherapy group stratified by stages I-IV. Corresponding group sizes and P‑values were presented. *P* < 0.05 was considered to indicate a statistically significant difference

Our analysis results indicated that the 4-year OS could potentially serve as a surrogate endpoint for patients with AJCC clinical stage I (80 vs. 78%, *P* = 0.250), regardless of the treatment received. As shown in Fig. 1a, the survival curves for stage I exhibited a smooth progression before reaching the 5-year mark.

In the chemoradiotherapy group, only the 3-year OS rate showed no significant difference compared to the 5-year rate for stage I patients (79 vs. 72%, *P* = 0.063). Figure 1b illustrated that the survival curve for stage I remained stable until it reached the 5-year mark, whereas the survival curve for locoregionally advanced disease (stage III-IV) displayed a downward trend until 7 or 8 years, resulting in markedly unsatisfactory survival compared to early stage cases (stage I-II). Similar trends were observed in the 1- to 5-year survival rates (Table 2).

**Fig. 2:**
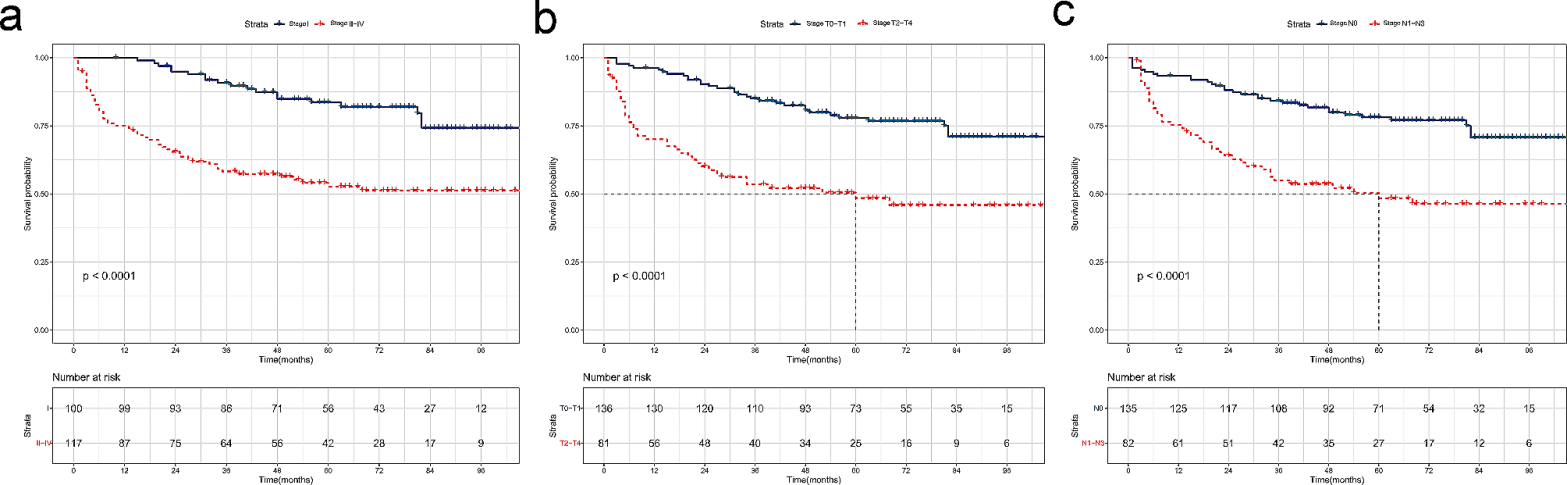
Radiotherapy group overall survival (OS) curves stratified by (**a**) stage I and II-IV; (**b**) stage T0-T1 and T2-T4; (**c**) stage N0 and N1-3. Corresponding group sizes and P‑values are presented. *P* < 0.05 was considered to indicate a statistically significant difference

Regarding the radiotherapy group, Fig. 1c revealed that due to the small size of stage II patients (41 patients), the survival curve remained indistinguishable after 5 years. Further division of the radiotherapy patients into specific subgroups (stage I and II-IV groups, T0-T1 and T2-T4 groups, N0 and N1-3 groups) for further analysis is presented in Fig. 2. For stage I, T0-T1, and N0 patients, the 4- and 5-year OS rates displayed no significant difference (83 vs. 82%, *P* = 1.000; 78 vs. 76%, *P* = 0.250;78 vs. 77%, *P* = 0.500, respectively). For stage II-IV, T2-T4, and N1-3 patients, the 3-year OS rates did not significantly differ from those at 5 years (57 vs. 51%, *P* = 0.063; 52 vs. 46%, *P* = 0.250;54 vs. 46%, *P* = 0.125, respectively).

## Discussion

Based on insights from the literature and the analysis of SEER data with its extensive sample size and long-term follow-up, we have successfully confirmed the viability of using short-term surrogate endpoints in evaluating 5-year OS outcomes for NPC patients in non-endemic areas.

Our analysis results indicated that the 4-year OS has the potential to serve as a suitable surrogate endpoint for patients with AJCC stage I NPC, irrespective of the treatment received. However, it is crucial to consider that different treatment strategies may influence survival outcomes, potentially impacting the study results. Therefore, we further categorized patients into radiotherapy and chemoradiotherapy groups to explore the surrogate endpoints. For patients who underwent chemoradiotherapy, the 3-year OS may act as a surrogate endpoint in stage I NPC. For those treated solely with radiotherapy, the 4-year OS may serve as a surrogate endpoint in stage I, T0-T1, and N0 NPC. Moreover, the OS rate at 3 years may serve as a surrogate endpoint for stage II-IV, T2-T4, and N1-N3 patients.

Selecting the primary endpoint for clinical trials remains a challenging task, and validated surrogate endpoints for OS play a crucial role in streamlining the clinical study and drug development processes. Nevertheless, relying on surrogates and intermediate endpoints bears the risk of trials that may not necessarily lead to improved long-term clinical outcomes. The potential biological mechanisms underlying the poor correlation of surrogate endpoints with OS are still not fully understood. From the perspective of clinicians, short-term overall survival is generally preferred and widely accepted.

The incidence of NPC exhibits significant regional variations, with Southern China reporting the highest rates [1, 8]. Studies and meta-analyses on NPC surrogate survival endpoints have predominantly been conducted in endemic areas with Chinese populations [18–23], which may limit the generalizability of these surrogate endpoints to regions with a low incidence of NPC. To the best of our knowledge, this study stands as the first of its kind to assess short-term OS as a surrogate endpoint of less than 5 years in non-endemic regions using the SEER database. The SEER is an authoritative database of cancer statistics with long follow-up data that allows more in-depth study of the prognosis and suitable treatment options. The SEER database gathers data from approximately one third of the United States population, offering valuable demographic, clinicopathologic, cancer incidence, and survival data [24, 25].

Chemoradiotherapy is considered the standard treatment for locally advanced NPC. However, a study by Liu et al. [26], using data from the SEER database, revealed significant effects of age, race, and tumor size on the death rate of patients with stage I-II NPC. For patients receiving chemoradiotherapy, the 3-year OS could serve as a surrogate endpoint for those in stage I. When comparing our findings with two similar studies focusing on high-incidence NPC areas [18, 19], we observed earlier OS surrogate endpoints in their studies. In one study, patients with stage I/II NPC received radiotherapy alone, while the other study did not specify the chemotherapy regimen, leaving the choice to the discretion of the clinicians. Both studies, interestingly, proposed using 1-year OS as a surrogate endpoint. This might be attributed to the fact that over 90% of Chinese patients are diagnosed with WHO III NPC, a cancer type associated with Epstein-Barr virus (EBV) [27]. On the other hand, about 40% of Western NPC cases fall under WHO I-II, which is more strongly associated with tobacco smoking [17, 28]. In our research, we observed that differential non-keratinizing carcinoma (WHO II, 29%) and keratinizing squamous cell carcinoma (WHO I, 33.3%) were the prevalent subtypes in the non-endemic region, which aligns with existing literature [29]. On the other hand, undifferentiated non-keratinizing carcinoma (WHO III) NPC is considered to exhibit more aggressive characteristics and an increased likelihood of distant metastasis, while cases classified as WHO I-II have been reported to be less responsive to treatment [30, 31]. Interestingly, WHO III NPC showed improved survival, which can be attributed, at least in part, to the tumor’s response to treatment and the earlier age of onset [32].

Figure 1B showed that the survival curves for stage I and II patients in the chemoradiotherapy group were initially closely aligned and only began to separate after the 5-year mark. Interestingly, beyond the 5-year mark, stage II patients showed slightly better survival rates compared to stage I patients. This difference in outcomes might be attributed to the potentially harmful effects of chemotherapy toxicities and delayed curative radiotherapy on treatment effectiveness. However, the association between induction chemotherapy, concurrent, and adjuvant chemotherapy with treatment outcomes for stage II NPC remains uncertain due to limited available data [33]. Furthermore, we observed a gradual convergence of the survival curves for stage II and III patients after 5 years. This suggests a substantial tumor heterogeneity among stage II and III patients, urging further investigation into the underlying biological mechanisms driving these variations in results.

For patients treated with radiotherapy, our findings suggest that the 4-year OS for stage I, T0-T1, and N0 NPC patients, as well as the 3-year OS for stage II-IV, T2-T4, N1-N3 NPC patients, could effectively serve as surrogate endpoints for the 5-year OS in NPC patients. Stage I and II-IV, T0-T1 and T2-T4, N0 and N1-3 represent two extremes regarding survival outcomes, with the surrogate endpoint for stage I, T0-T1, and N0 indicating stable and favorable treatment effects, and that for stage II-IV, T2-T4, N1-N3 indicating stable but poorer treatment outcomes. Both stages exhibited a rapid plateau in survival, likely influenced by the utilization of intensity-modulated radiotherapy (IMRT), which is known to be superior to two-dimensional conventional radiation therapy (2D-CRT) [34]. Patients treated between 2010 and 2015 were included in this study. The United States was the first country to develop IMRT technology in 1995, which was subsequently promoted worldwide [35]. However, due to limitations in the SEER database, specific details regarding the radiotherapy techniques used in the included patients could not be extracted.

Our analysis sheds valuable light on the concept of using short-term surrogate endpoints (less than 5 years) for a cohort of NPC patients in a non-endemic area, which presents several differences from cohorts in high-incidence areas. This contributes significantly to the global significance of our findings and offers promising prospects for medical professionals by potentially shortening the time required for therapeutic evaluation, conserving medical resources, and expediting future clinical trials and the development of effective treatment approaches for non-endemic NPC patients. Embracing short-term OS as surrogate endpoints could pave the way for more individualized and cost-effective surveillance for cancer survivors, benefiting post-treatment follow-up practices for cancer survivors.

Nevertheless, it is vital to acknowledge the study’s limitations. First, due to its retrospective nature, inherent bias might influence the results, and the lack of external validation is a point of consideration. Second, the majority of our study patients received chemoradiotherapy, possibly introducing bias toward outcomes. Moreover, the limited number of patients receiving radiotherapy and the absence of further stratification by the AJCC staging system could restrict the generalizability of our conclusions. Third, the SEER database does not provide specific details on radiotherapy techniques and chemotherapy regimens, coupled with the potential application of new treatment strategies, which might have led to more optimal patient survival, potentially resulting in an underestimation of the calculated survival data. Fourth, the absence of crucial information such as smoking, alcohol consumption, and nutritional status, which may correlate with prognosis, adds a layer of complexity to the interpretation. Additionally, the SEER database does not contain data on NPC recurrence and metastasis after treatment, hindering the evaluation of other survival surrogate endpoints.

## Conclusion

Our study advocates for the potential use of short-term surrogate endpoints in evaluating the 5-year overall survival outcomes for NPC patients in non-endemic areas. However, further research is imperative to validate and reinforce these findings.

### Electronic supplementary material

Below is the link to the electronic supplementary material.

Supplementary Material 1

## Data Availability

Publicly available datasets were analyzed in this study. This data can be found here: https://seer.cancer.gov/data/.
