# Supplementary material for: Short-term OS as a surrogate endpoint for 5-year OS in nasopharyngeal carcinoma in non-endemic area
Source: World J Surg Oncol. 2024 Jul 11;22:180. doi: 10.1186/s12957-024-03460-z (PMC11238357; doi:10.1186/s12957-024-03460-z)
Supplement: Supplementary file 1 — Supplementary Material 1 [file 12957_2024_3460_MOESM1_ESM.docx]

**Supplementary Information**

**Table S1.** Summary of literatures on surrogate survival endpoints for NPC.

| Reference | Study design and Type | Number and stage of patients | Treatment | Radiotherapy technique | Less than 5-year Substitute endpoints |
| --- | --- | --- | --- | --- | --- |
| Chen C ^[18]^ 2013 et al. | Retrospective  Single center | ^#^Total :2450  Stage I:127  Stage II:864  Stage III:986  Stage IV:473 | RT alone: Stage I/II  RT+CT: Stage III/IV  Total RT dose: 60-78Gy^₡^ | 2D-RT:1842  3D-RT:451  IMRT:157  Stage I:16  Stage II:34  Stage III:72  Stage IV:35 | For 2D RT: Stage I:1-y OS and 1-y LRC  N3:4-y OS and 2-y LRC  For IMRT: Stage I/II:1-y OS and 1-yLRC  Stage III/IV:4-y OS and 4-yLRC |
| Zhou S ^[19]^ 2018 et al. | Retrospective  Single center | ^€^Total :830  Stage I:18  Stage II:129  Stage III:423  Stage IVA:260 | Total RT dose: ≥66Gy, 2.0-2.27Gy per fraction^₡^  Chemotherapy regimen：determined by the clinicians ^₡^ | IMRT | Total patients:  Stage I: 1y-OS, PFS, LRFS, DMFS; Stag II: 3y-OS, PFS, LRFS, 4y-DMFS; Stage III: 4y-LRFS, DMFS; Stage IVA: 3y-LRFS, DMFS |
|  |  |  |  |  | Low EBV patients:  Stage I: 1y-OS, PFS, LRFS, DMFS; Stag II: 3y-OS, PFS, LRFS, 4y-DMFS; Stage III and IVA: 4y-PFS, 3y-LRFS, DMFS |
|  |  |  |  |  | Mid EBV patients:  Stage I: 1y-OS, PFS, LRFS, DMFS; Stag II: 3y-OS, 2y-PFS, 2y-LRFS, 1y-DMFS; Stage III: 4y-OS, PFS, LRFS, DMFS; Stage IVA: 4y-OS, 3y-PFS,3y-LRFS, 2y-DMFS |
|  |  |  |  |  | High EBV patients:  Stag II: 1y-OS, 4y-PFS, 4y-LRFS, 1y-DMFS; Stage III: 3y-OS, 3y-PFS, 2y-LRFS, 4y-DMFS; Stage IVA: 4y-OS, 4y-PFS, 3y-LRFS, 3y-DMFS |
| Chen YP ^[20]^ 2015 et al. | Prospective  Phase III randomized clinical trial  Single center | ^∞^Stage III-IVB (T3-4NxM0 or TxN2-3M0): 316 | IMRT alone group(n=158): Total RT dose:＞ 66Gy^₡^  versus CRT group(n=158)：IMRT+  7×DDP 40mg/m^2^/w(day 1)+3×PF：cisplatin 80mg/m^2^/d (day 1) +fluorouracil 800mg/m^2^/d (days 1-5 civ) | 2D-RT | Secondary results of clinical trial  Substitute for 5-year OS: 2-y, 3-y PFS and FFS, independently of treatment |
| Chen PL ^[21]^ 2015 et al. | Retrospective | ^#^Stage III:136  Stage IVA-IVB:72 | Total RT dose:68-76Gy^₡^  2-2.27Gy per fraction  CCRT alone group(n=104): cisplatin-based  versus NACT+CCRT group(n=108)：  PF | IMRT | Substitute for 5-year OS: 3-y PFS, FFS and D-FFS, 3-y PFS maybe the most accurate |
| Chen YP ^[22]^ 2015 et al. | Meta-analysis | Total: 5212 | 21 combined treatment-control comparisons trials (1997-2007)  FFS:16 trials  PFS:9 trials | N/A | Substitute for 5-year OS: 3-y PFS and FFS, PFS may be a more acceptable surrogate endpoint compared with FFS |
| Rotolo F ^[23]^ 2017 et al. | Meta-analysis | Total: 5144 | 20 chemotherapy trials for loco-regionally advanced NPC trials (1998-2010)  N/A | N/A | Substitute for 5-year OS: 2-y PFS and DMFS, while PFS can be measured earlier |

#: Restaged according to the Union for International Cancer Control(UICC) 2002 staging system; ₡: Not speciﬁed;€: Restaged according the 8th edition of the UICC/AJCC staging system; CRT: concurrent chemoradiotherapy with adjuvant chemotherapy; **∞:** disease according to the 1997 American Joint Commission on Cancer (AJCC) staging system;2D-RT: 2-dimensional radiotherapy; 3D-RT:3-dimensional conformal radiotherapy; IMRT: intensity-modulated radiotherapy; EBV: Epstein-Barr Virus; NPC: Nasopharyngeal Carcinoma; OS: overall survival; LRC: locoregional control ; PFS: progression-free survival; LRRFS/LRFS: loco-regional recurrence-free; DMFS: distant metastasis-free survival; FFS: failure-free survival; D-FFS: distant metastasis-free failure survival; LR-FFS : locoregional failure free survival; DDP: cisplatin; N/A: not available.
